# Integrated pharmacovigilance of fentanyl: multinational analysis reveals novel safety signals and demographic-specific risk profiles

**DOI:** 10.3389/fphar.2025.1700112

**Published:** 2025-10-08

**Authors:** Junge Zhang, Wenxin Wang, Zihui Lu, Changshun Huang, Hehe Wang

**Affiliations:** ^1^ Department of Anesthesiology, the First Affiliated Hospital of Ningbo University, Ningbo, China; ^2^ Department of Otolaryngology, Head and Neck Surgery, the First Affiliated Hospital of Ningbo University, Ningbo, China

**Keywords:** fentanyl, pharmacovigilance, adverse drug events, signal detection, opioid safety

## Abstract

**Background:**

This study aimed to characterize the comprehensive safety profile of fentanyl, including emerging and demographic-specific risks, by analyzing international pharmacovigilance data.

**Methods:**

We conducted a retrospective analysis of fentanyl-associated adverse drug events (ADEs) from the FDA Adverse Event Reporting System (FAERS) and the Japanese Adverse Drug Event Report database (JADER) (2004–2025). Data integration was followed by rigorous cleaning, standardization using MedDRA v27.0, and multi-method disproportionality analysis (ROR, PRR, BCPNN, MGPS). Sensitivity analyses and time-to-onset modeling were performed to evaluate temporal risk dynamics.

**Results:**

Among 76,903 reports, 396 significant signals were detected in FAERS and 95 in JADER. FAERS emphasized behavioral and device-related events (e.g., drug abuse [ROR = 31.3], administration errors [ROR = 71.08]), while JADER captured acute physiological events (e.g., respiratory depression [ROR = 57.41], neonatal respiratory failure [ROR = 212.77]). Novel signals included Kounis syndrome, application site injuries, and neonatal withdrawal. Gender disparities showed higher risks of administration errors and application site reactions in females, and misuse and overdose in males. Most events (51%) occurred within 1 month of treatment initiation.

**Conclusion:**

Fentanyl’s risk profile varies significantly across regions and demographics, influenced by reporting biases and clinical use patterns. These findings advocate for global harmonization of surveillance practices and targeted risk mitigation strategies.

## 1 Introduction

Fentanyl, a potent synthetic μ-opioid receptor agonist, remains indispensable for managing severe acute and chronic pain in oncologic, perioperative, and palliative settings. Its high lipophilicity, rapid blood-brain barrier penetration, and versatile delivery systems—including transdermal patches, injectables, and buccal formulations—enable tailored analgesia for diverse clinical needs (Mahinthichaichan et al., 2021; Grape et al., 2010). Yet these very properties also introduce critical safety challenges: a narrow therapeutic index, a significant potential for misuse, and the risk of life-threatening respiratory depression. These characteristics have placed fentanyl at the center of overlapping public health crises, contributing to the steady rise in opioid-related overdose deaths in the United States since 2000 (Rudd et al., 2016a; Rudd et al., 2016b; Fitzgerald et al., 2025a; Simha et al., 2023). Furthermore, contemporary pharmacovigilance must address not only established opioid toxicities but also emergent, device-dependent, and demographic-specific risks identified through real-world utilization patterns.

Spontaneous reporting systems (SRS) provide essential infrastructure for detecting rare or delayed adverse drug events (ADEs). This study leverages two complementary global pharmacovigilance databases. The primary data source is the FDA Adverse Event Reporting System (FAERS), a cornerstone surveillance database that aggregates over 9 million voluntary reports from healthcare professionals, consumers, and manufacturers. FAERS integrates seven interlinked datasets—Demographics (DEMO), Drug Information (DRUG), Adverse Reactions (REAC), Patient Outcomes (OUTC), Report Sources (RPSR), Therapy Dates (THER), and Indications (INDI)—using unique CASEID identifiers (Noguchi et al., 2021; Potter et al., 2025). We supplement the FAERS data with reports from Japan’s Pharmaceuticals and Medical Devices Agency (PMDA) Adverse Drug Event Report database (JADER). The JADER database, which has collected institutional and manufacturer reports since 2004, is structured around four core datasets: DEMO, DRUG, REAC, and Patient History (HIST) (Nomura et al., 2015).

Despite their utility, inherent limitations constrain these systems: geographic reporting biases (e.g., FAERS overrepresents U.S. reports; JADER reflects Japanese prescribing patterns), heterogeneous MedDRA coding practices, and signal fragmentation when analyzed in isolation (Tsuchiya et al., 2020; Fouretier et al., 2016). Integrating FAERS and JADER offers a strategic approach to mitigate these constraints, enhancing statistical power while enabling cross-population signal validation—particularly salient for fentanyl given its formulation diversity and regional prescribing variations (Lou et al., 2025).

Current evidence inadequately characterizes emerging safety concerns beyond classical opioid ADEs, including device-specific complications with transdermal systems, neonatal morbidity following maternal exposure, severe autonomic crises, and demographic-stratified vulnerabilities (Armenian et al., 2018). Furthermore, temporal hazard dynamics and geography-dependent risk patterns remain poorly defined, limiting precision risk mitigation (Chiappini et al., 2022).

**FIGURE 1:**
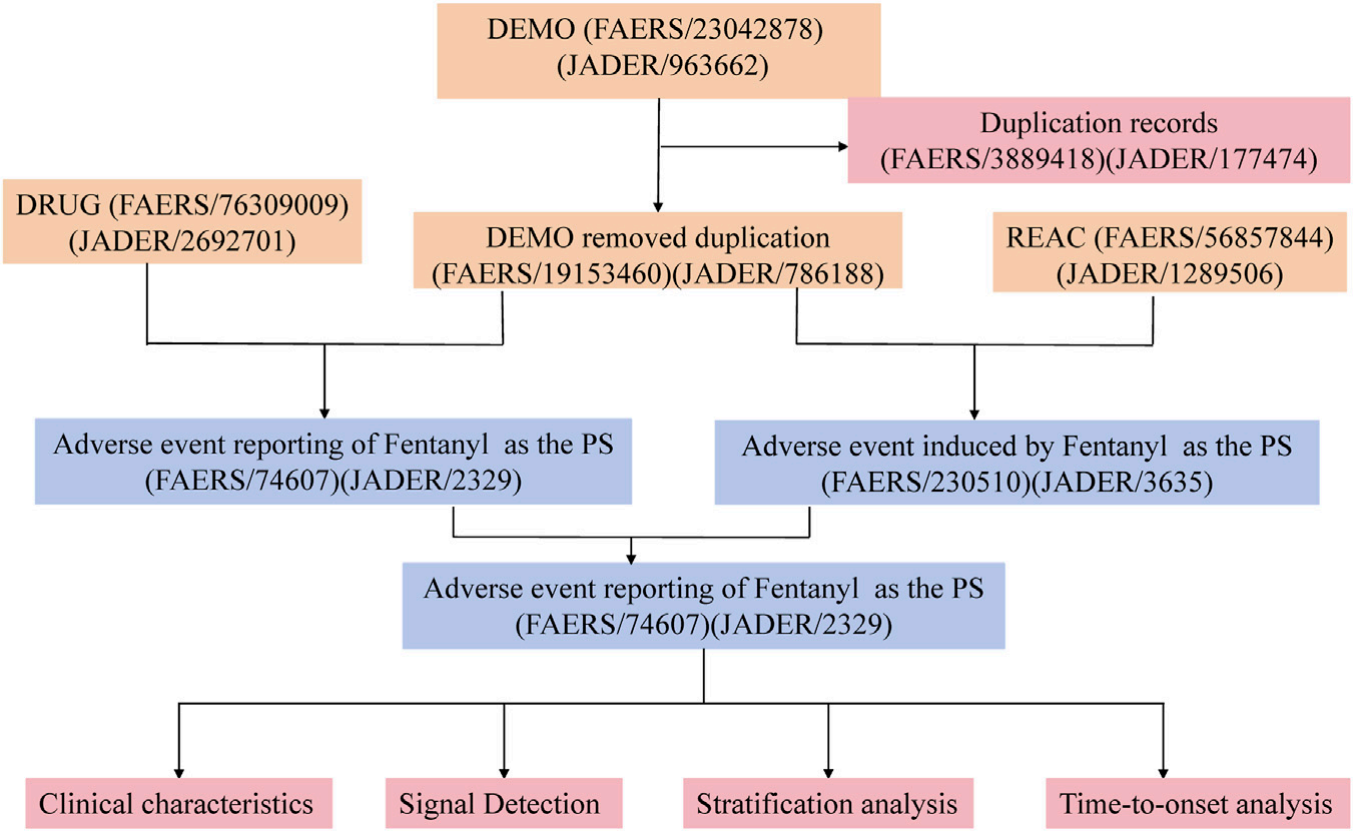
The flowchart of this study.

To address these gaps, we employ an integrated pharmacovigilance framework harmonizing FAERS and JADER data (2004–2025) with multi-algorithm disproportionality analysis (ROR, PRR, BCPNN, MGPS) and time-to-onset modeling. Our objectives are threefold: (1) characterize the spectrum and strength of fentanyl-associated ADEs across international surveillance systems; (2) identify potentially novel signals absent from current labeling; and (3) elucidate demographic-specific and temporal risk patterns to inform targeted risk management strategies. Preliminary findings of device-dependent toxicities and neonatal complications underscore the urgency of this comprehensive safety assessment. The process of this research is shown in the flowchart (Figure 1).

## 2 Materials and methods

### 2.1 Data sources and collection

In this study, we performed a retrospective pharmacovigilance analysis using data from the FDA Adverse Event Reporting System (FAERS) and the Japanese Adverse Drug Event Report database (JADER) to evaluate adverse drug events (ADEs) associated with fentanyl. Both databases represent large-scale spontaneous reporting systems that play a crucial role in detecting potential safety signals in the post-marketing setting. To improve statistical power and minimize geographical reporting biases that may affect individual databases, we combined data from both FAERS and JADER for a more comprehensive safety evaluation of fentanyl. The analysis included FAERS data from the first quarter of 2004 to the second quarter of 2025 and JADER data covering the same period, from Q1 2004 to Q2 2025. We identified fentanyl-related reports by comprehensively searching the DRUG files using the generic name (“Fentanyl”), relevant chemical denominations, and major brand names (e.g., “Sublimaze,” “Duragesic,” “Actiq”). For JADER, search terms included the Japanese generic name “フェンタニル” as well as relevant brand names (e.g., “フェンタネスト”).

To ensure analytical rigor, a robust data cleaning process was applied. Duplicate case reports were identified and removed in accordance with standard FDA guidelines. Using the unique CASEID and the FDA receipt date (FDA_DT in FAERS; analogous fields in JADER), the most recent version of each report was retained to preserve the latest and most comprehensive information for each case. All adverse event entries were standardized using Medical Dictionary for Regulatory Activities (MedDRA) terminology, version 27.0. Within the MedDRA hierarchy, adverse events were classified into Preferred Terms (PTs) and System Organ Classes (SOCs). The analysis was restricted to reports in which fentanyl was reported as the “Primary Suspect” (PS) drug, thereby focusing on cases with the strongest temporal and causal likelihood of association.

### 2.2 Signal detection and statistical analysis

To evaluate potential safety signals associated with fentanyl, we employed a comprehensive disproportionality analysis framework using four distinct algorithms: the Reporting Odds Ratio (ROR) (Rothman et al., 2004), Proportional Reporting Ratio (PRR) (Evans et al., 2001), Bayesian Confidence Propagation Neural Network (BCPNN) (Bate et al., 1998), and Multi-Item Gamma Poisson Shrinker (MGPS) (Szarfman et al., 2002). The frequentist methods (ROR and PRR) served as initial measures of association, with ROR selected for its reduced sensitivity to sparse data and PRR for its relative stability in the context of underreporting. Bayesian approaches (BCPNN and MGPS) were applied to incorporate formal uncertainty quantification; BCPNN facilitated evidence synthesis across multiple dimensions, while MGPS enhanced detection capability for rare adverse events. This multi-method strategy strengthens signal detection by leveraging complementary statistical properties across frequentist and Bayesian paradigms.

To minimize false positives often arising from single-method disproportionality analyses, we applied a stringent consensus criterion: only signals meeting the predefined statistical thresholds in all four algorithms were considered significant associations. Control over type I error inflation was achieved using Bonferroni correction for multiple testing. All statistical analyses were performed using R version 4.3.3 (R Foundation for Statistical Computing), with implemented disproportionality methods drawn from specialized pharmacovigilance packages.

### 2.3 Time to onset analysis

The time to onset (TTO) of fentanyl-associated adverse drug events was defined as the duration from therapy initiation (START_DT in THER) to the occurrence of the event (EVENT_DT in DEMO). Cases with incomplete or implausible temporal information—such as missing dates or dates lacking day, month, or year specificity—were excluded. Reports in which the documented event preceded the start of fentanyl were also removed to avoid negative TTO values.

Descriptive statistics—including median, interquartile range, minimum, and maximum—were used to summarize TTO. The cumulative incidence of events over time was estimated using Kaplan-Meier analysis. To model the hazard function, a Weibull distribution was fitted, and the shape parameter (β) was interpreted to assess risk dynamics over time. Consistent with pharmacoepidemiologic convention, a β < 1 (with 95% confidence interval excluding 1) was interpreted as indicating a decreasing hazard; β ≈ 1 (95% CI including 1) as constant hazard; and β > 1 (95% CI excluding 1) as increasing hazard.

## 3 Results

### 3.1 Descriptive characteristics

**FIGURE 2:**
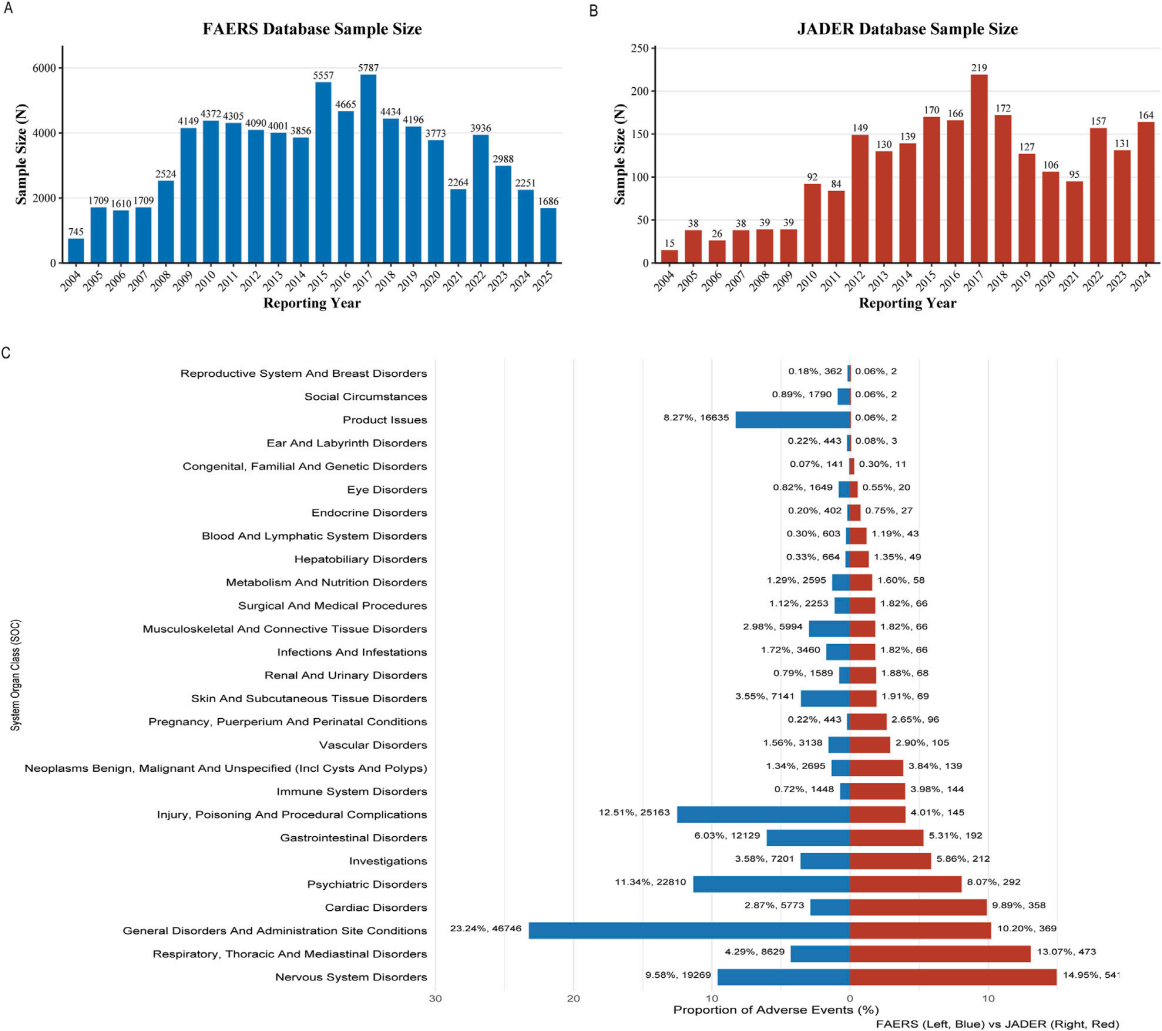
Descriptive characteristics. **(A)** Annual number of reports of fentanyl-associated adverse events in the FAERS database from Q1 2004 to Q2 2025; **(B)** Annual number of reports of fentanyl-associated adverse events in the JADER database from Q1 2004 to Q2 2025; **(C)** The SOC distribution map of FAERS and JADER.

Analysis of fentanyl-related ADEreports identified 74,607 cases in the FAERS (Figure 2A) and 2,296 cases in JADER (Figure 2B) database from the first quarter of 2004 through the second quarter of 2025. Both systems exhibited similar temporal trends, characterized by an initial increase in reporting frequency, a peak in 2017, and a subsequent decline. Evaluation of System Organ Class (SOC) distributions revealed distinct profiles between the two databases. In FAERS, the most commonly reported SOC categories included general disorders and administration site conditions, injury‒poisoning and procedural complications, and psychiatric disorders. In contrast, JADER was dominated by nervous system disorders, respiratory‒thoracic and mediastinal disorders, and general disorders and administration site conditions (Figure 2C).

Demographic characteristics are summarized in Table 1. In FAERS, females accounted for a higher proportion of reports (47.9%; *n =* 35,753) than males (40.0%; *n =* 29,858). The largest age group was 18–64 years (*n =* 28,133; 37.7% of reports with age information). Weight was not documented in 72.0% of cases (*n =* 53,716); among those with available data, the majority of patients (21.3%) weighed between 50 and 100 kg. Consumers were the most frequent reporters (45.3%; *n =* 33,773). Among all reports, serious outcomes were common, including hospitalization (13.5%; *n =* 10,061) and death (24.0%; *n =* 17,928). The United States, Japan, and France were the primary reporting countries. Within JADER, reports involved 47.4% males (*n =* 1,088) and 45.9% females (*n =* 1,055). Patients between 20 and 69 years of age constituted 48.4% (*n =* 1,111) of cases with age documentation. Where weight was reported (21.6%; *n =* 495), most patients fell within the 50–100 kg range. Physicians submitted the majority of reports (54.7%; *n =* 1,256).

**TABLE 1:** Characteristics of fentanyl from FAERS and JADER databases.

Characteristics, number (%)	FAERS	JADER
Number of events	74,607	2,296
Gender		
Male	29,858 (40.0%)	1,088 (47.4%)
Female	35,753 (47.9%)	1,055 (45.9%)
Unknown	8,996 (12.1%)	153 (6.7%)
Age (years)		
<2	543 (0.7%)	<10,212 (9.2%)
2∼11	526 (0.7%)	
12∼17	613 (0.8%)	10∼1,972 (3.1%)
18∼64	28,133 (37.7%)	20∼691,111 (48.4%)
65∼85	7,666 (10.3%)	>70,796 (34.7%)
>85	1,294 (1.7%)	
Unknown	35,832 (48.0%)	105 (4.6%)
Weight (Kg)		
<50 kg	1822 (2.4%)	340 (14.8%)
50∼100 kg	15,900 (21.3%)	495 (21.6%)
>100 kg	3,169 (4.2%)	24 (1.0%)
Missing	53,716 (72.0%)	1,437 (62.6%)
Reported countries (Top3)		
United States of America	55,689 (74.6%)	Japan (100%)
Japan	4,017 (5.4%)	
France, French Republic	2,244 (3.0%)	
Reporter		
Physician	17,595 (23.6%)	1,256 (54.7%)
Pharmacist	9,615 (12.9%)	NA
Consumer	33,773 (45.3%)	NA
Health Professional	3,055 (4.1%)	NA
Missing	10,569 (14.2%)	40 (1.7%)
Outcome		
Congenital Anomaly	31 (0.0%)	NA
Death	17,928 (24.0%)	416 (11.4%)
Disability	474 (0.6%)	NA
Hospitalization	10,061 (13.5%)	NA
Life-Threatening	1,129 (1.5%)	NA
Other	44,984 (60.3%)	NA

### 3.2 Signal detection at the PT level

Our multi-method disproportionality analysis identified 396 and 95 significant safety signals associated with fentanyl in the FAERS and JADER databases, respectively. To enhance clinical interpretability, we focus here on the most salient signals—selected based on their high frequency, exceptional signal strength (ROR), clinical severity, and novelty—while the complete listings are provided in Supplementary Tables S1, S2, S4.

**FIGURE 3:**
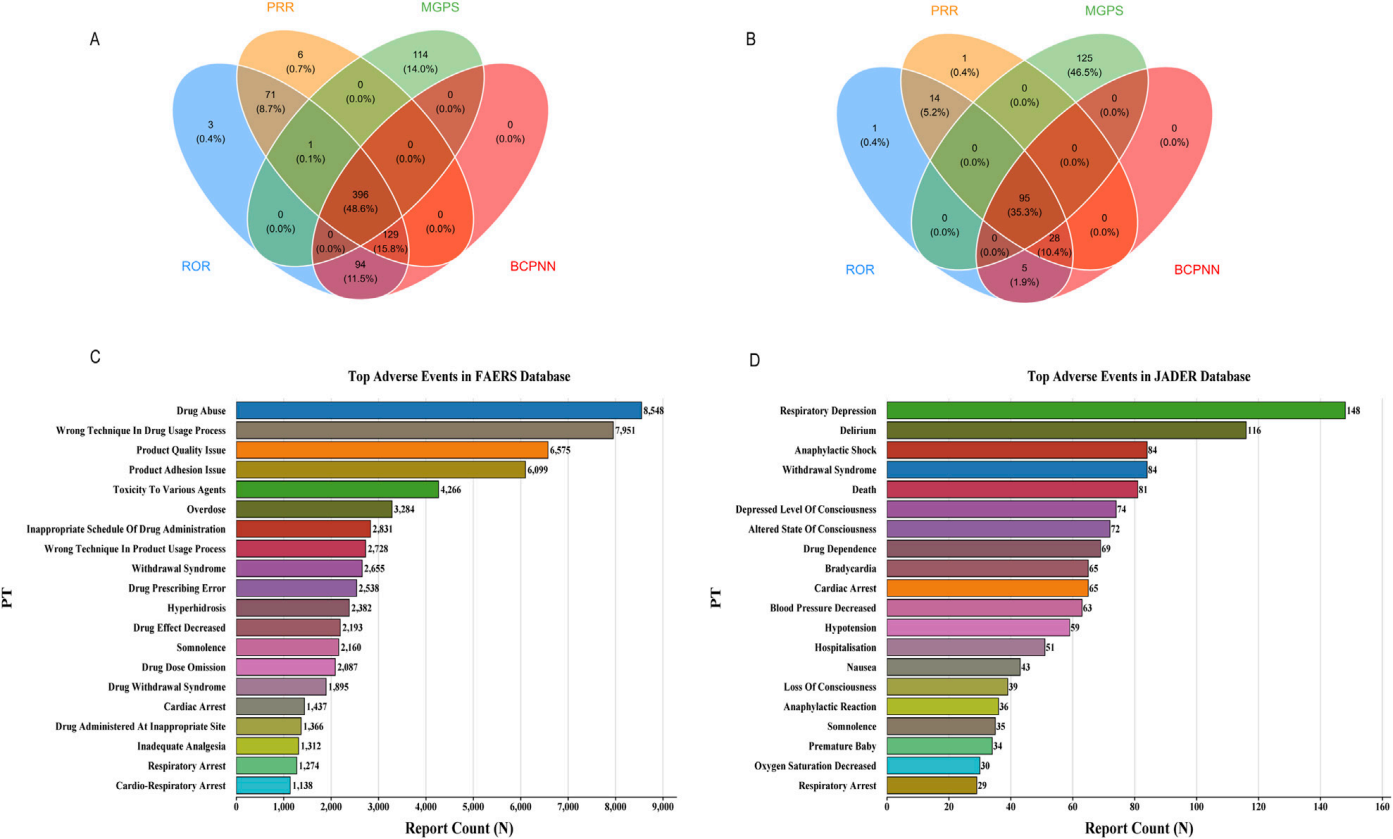
Signal detection at the PT level. **(A)** 396 significant signals in FAERS; **(B)** 95 significant signals in JADER; **(C)** In FAERS, the most frequently detected signals included drug abuse, incorrect technique in drug usage process, and product quality issue; **(D)** The most prominent signals in JADER were respiratory depression, delirium, and anaphylactic shock.

The overall signal profiles diverged markedly between the two databases (Figures 3A,B). FAERS was characterized by a high frequency of reports related to drug abuse, incorrect technique in drug usage process, and product quality issues (Figure 3C). In contrast, JADER was dominated by acute physiological events, with respiratory depression, delirium, and anaphylactic shock being the most prominent (Figure 3D).

**FIGURE 4:**
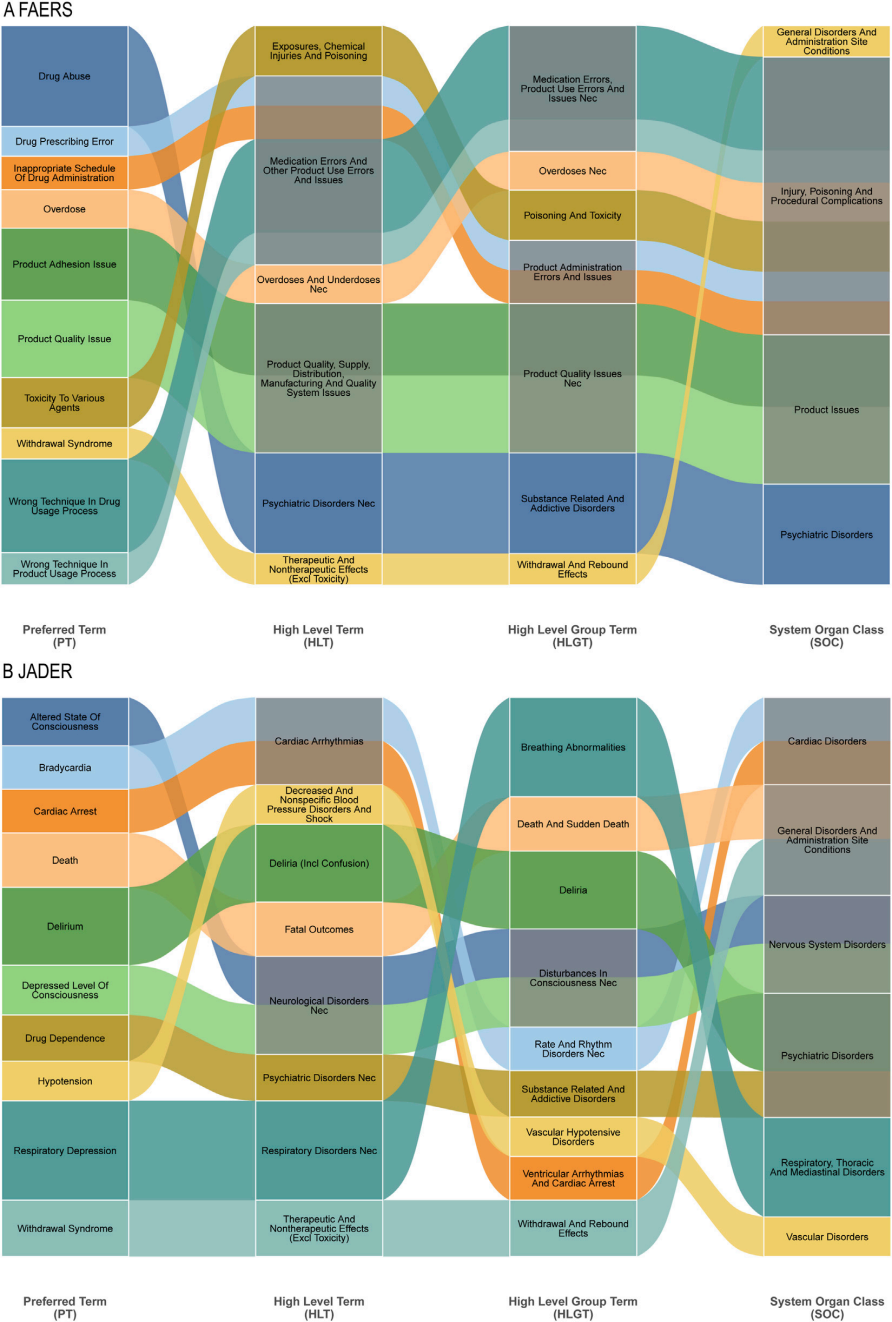
Top 30 positive signals simultaneously meeting the criteria of all four algorithms. Signals are ranked by Reporting Odds Ratio (ROR) with 95% confidence intervals. The four disproportionality analysis methods used were: Reporting Odds Ratio (ROR), Proportional Reporting Ratio (PRR), Bayesian Confidence Propagation Neural Network (BCPNN), and Multi-item Gamma Poisson Shrinker (MGPS). A signal was considered significant only if it met the predefined thresholds in all four methods. **(A)** Top 30 positive signals in FAERS. **(B)** Top 30 positive signals in JADER.

A detailed examination of the top signals, visualized by their Reporting Odds Ratios (RORs) in Figures 4A,B, revealed critical findings. In FAERS, several signals combined high report counts with strong disproportionality, underscoring substantial public health impact. These included drug abuse (*n =* 8,548, ROR = 31.3), wrong technique in drug usage process (*n =* 7,951, ROR = 71.08), and product quality issue (*n =* 6,575, ROR = 13.62). Notably, we also detected potent signals with lower frequency but extreme association strength, such as narcotic intoxication (ROR = 232.96) and breakthrough pain (ROR = 94.06).

Critically, the analysis uncovered novel signals not currently highlighted in fentanyl prescribing information. In FAERS, these predominantly pertained to complications of transdermal delivery systems, including product adhesion issues and various application site reactions (e.g., pain, burn, erosion). In JADER, the novel signals indicated severe and previously under-recognated clinical syndromes, most notably neonatal respiratory failure (ROR = 212.77), negative pressure pulmonary edema (ROR = 218.17), and severe autonomic crises such as Kounis syndrome.

**FIGURE 5:**
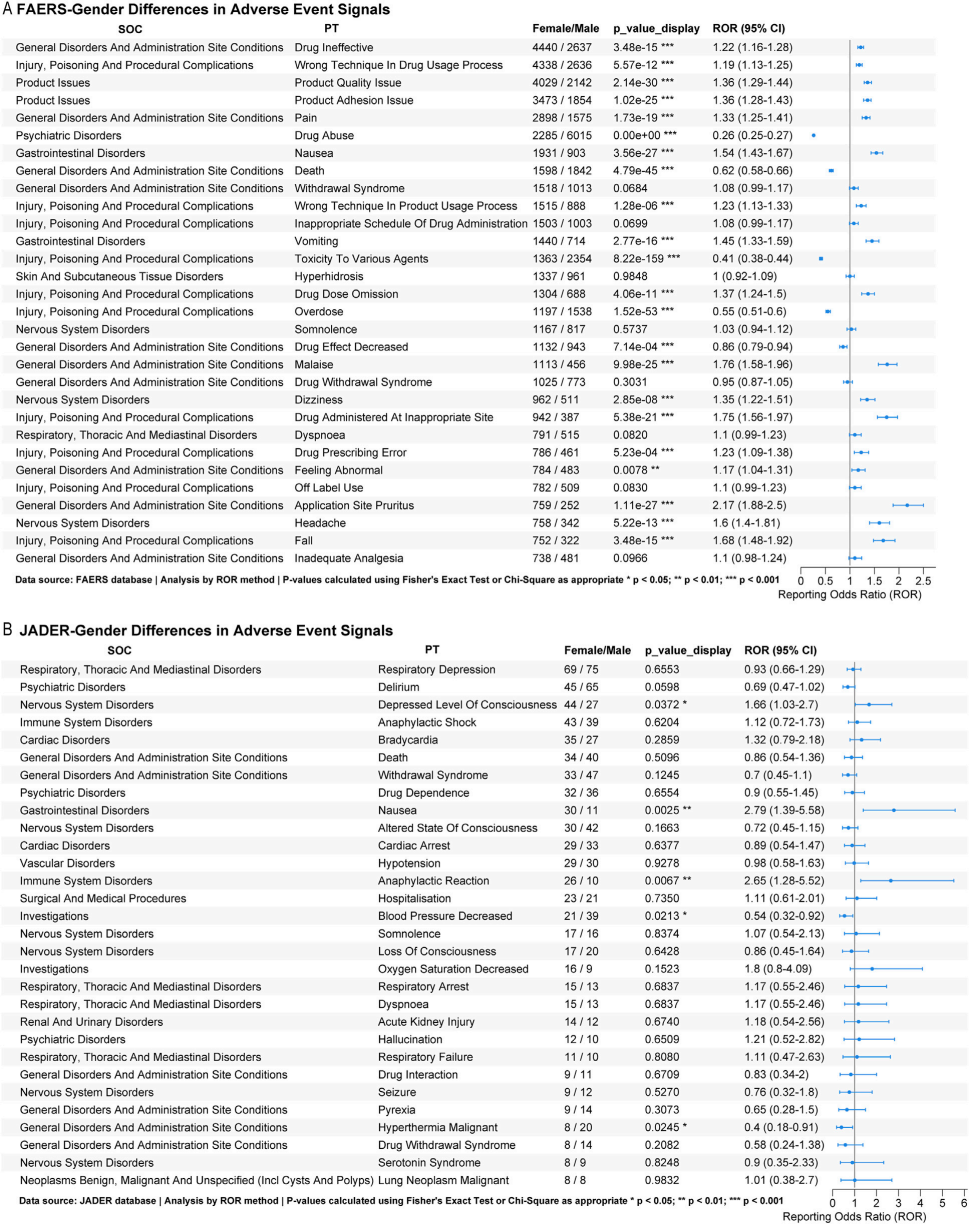
Sankey Diagram: Mapping of specific adverse events at the Preferred Term (PT) level to their broader MedDRA classifications for fentanyl-associated reports. **(A)** Top 10 PTs in FAERS and their connections to higher-level term groupings. **(B)** Top 10 PTs in JADER and their connections to higher-level term groupings.

Hierarchical clustering analysis further crystallized the fundamental differences in pharmacovigilance priorities between the systems (Figures 5A,B). In FAERS, signals aggregated under High-Level Terms (HLTs) like “Substance-related disorders” and “Medication errors,” reflecting concerns around misuse and medication safety. Conversely, JADER signals were concentrated in HLTs such as “Breathing abnormalities” and “Disturbances in Consciousness,” indicating a primary focus on acute organ system toxicities. This structural divergence underscores that FAERS captures themes of public health and regulatory concern, while JADER provides a more granular view of direct physiological compromise.

### 3.3 Subgroup analysis

**FIGURE 6:**
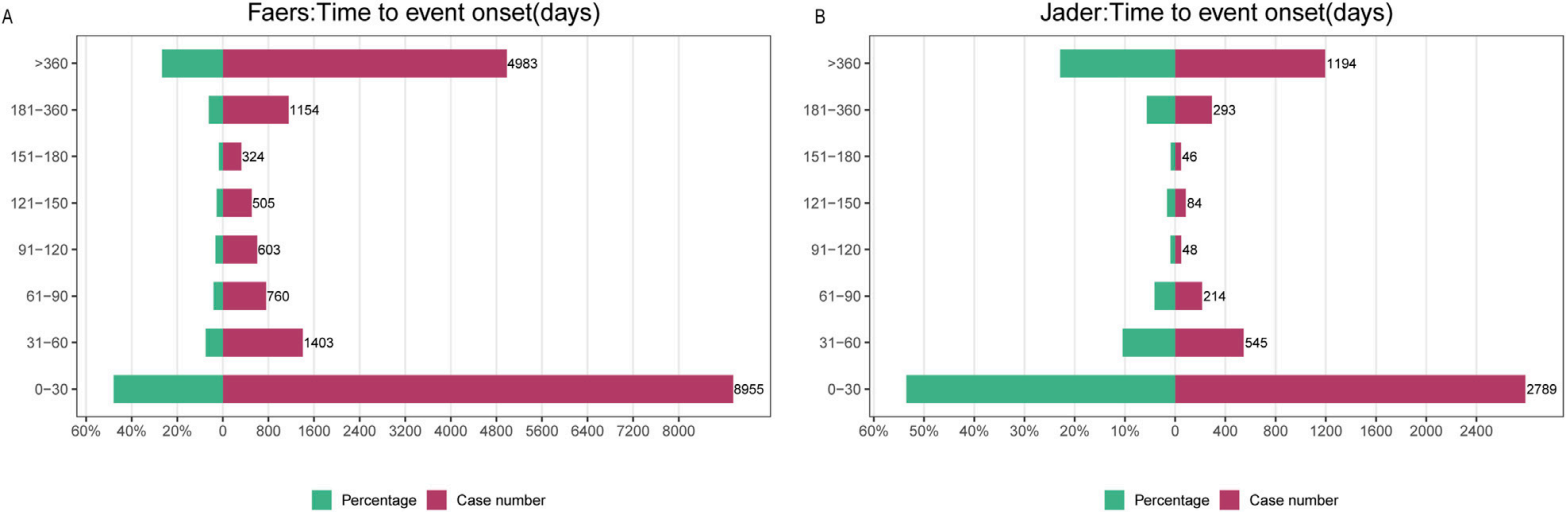
Subgroup analysis. **(A)** Sex-based subgroup analysis of the top 30 fentanyl-related adverse events in FAERS; **(B)** Sex-based subgroup analysis of the top 30 fentanyl-related adverse events in JADER.

Our pharmacovigilance analysis identified significant gender-based differences in fentanyl-associated adverse events across international databases. In FAERS, females showed significantly higher reporting odds (as measured by ROR) for therapeutic failure (drug ineffective: ROR = 1.22, p < 0.001), administration errors (wrong technique: ROR = 1.19, p < 0.001), product quality concerns (product quality issue: ROR = 1.36; product adhesion issue: ROR = 1.36; both p < 0.001), and gastrointestinal events (nausea: ROR = 1.54, p < 0.001). In contrast, reports for males were significantly more likely to experience substance misuse (drug abuse: ROR = 0.26, p < 0.001) and overdose-related outcomes (poisoning: ROR = 0.41; overdose: ROR = 0.55; both p < 0.001). Females also had a higher frequency of reports for application site reactions (pruritus: ROR = 2.17, p < 0.001) and procedural complications (Figure 6A). Conversely, JADER data revealed a distinct profile: Japanese females were associated with significantly higher reporting odds for impaired consciousness (depressed level of consciousness: ROR = 1.66, p = 0.037), gastrointestinal distress (nausea: ROR = 2.79, p = 0.003), and hypersensitivity reactions (anaphylactic reaction: ROR = 2.65, p = 0.007), but lower reporting for decreased blood pressure (ROR = 0.54, p = 0.021) compared to males (Figure 6B).

These findings indicate consistent gender-specific safety profiles across geographic regions: females demonstrate greater vulnerability to product- and administration-related issues and gastrointestinal or hypersensitivity events, whereas males are more susceptible to intentional misuse and serious overdose outcomes.

### 3.4 Time to onset analysis

**FIGURE 7:**
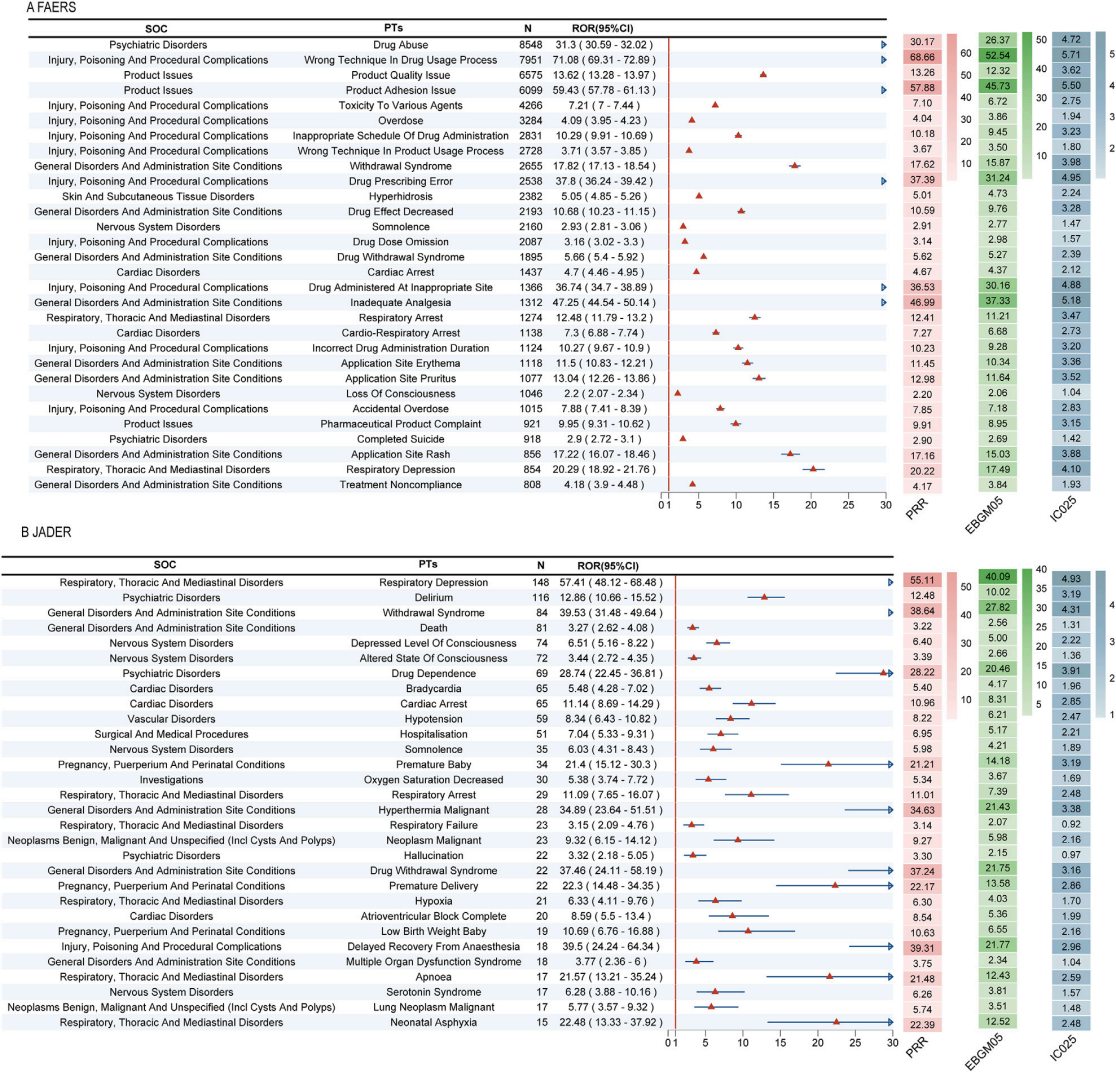
Time to onset analysis. **(A)** Time to onset of fentanyl-associated adverse events in FAERS; **(B)** Time to onset of fentanyl-associated adverse events in JADER.

Analysis of fentanyl-associated adverse drug events (ADEs) revealed distinct temporal patterns across pharmacovigilance databases. In FAERS, the majority of ADEs (51%; *n =* 2,481) occurred within 1 month of initiation, while a substantial proportion (23.1%; *n =* 1,124) emerged after more than 1 year. Reporting rates declined progressively during the first 180 days but stabilized thereafter, consistently accounting for approximately 30% of all cases (Figure 7A). Weibull distribution modeling indicated a decreasing hazard profile over time, supported by a shape parameter β = 0.43 (95% CI < 1) (Table 2). A similar time-dependent pattern was observed in JADER, which exhibited comparable hazard dynamics and reporting chronology (Figure 7B).

**TABLE 2:** Weibull distribution model parameters for the time to onset analysis.

	Parameter	Estimate	CI_lower	CI_upper	KS_pvalue	AD_pvalue
FAERS	shape (β)	0.43	0.421	0.439	0	1.23E-07
	scale (λ)	111.77	104.03	119.52	0	1.23E-07
JADER	shape (β)	0.533	0.522	0.544	0	1.15E-07
	scale (λ)	90.01	85.15	94.87	0	1.15E-07

CI_lower, CI_upper: Lower and upper bounds of the 95% confidence interval. KS_pvalue, AD_pvalue: P-values of the Kolmogorov-Smirnov and Anderson-Darling goodness-of-fit tests, respectively. A p-value <0.05 indicates a significant deviation from the Weibull distribution. The value ‘0’ denotes a p-value smaller than the computational precision. Interpretation of shape parameter (β): β < 1 indicates a decreasing hazard over time (i.e., highest risk immediately after treatment initiation); β = 1 indicates a constant hazard; β > 1 indicates an increasing hazard.

## 4 Discussion

This integrated analysis of international pharmacovigilance data elucidates three pivotal dimensions of fentanyl safety that extend beyond established opioid toxicology (Petrosky et al., 2018). First, the stark divergence in adverse event profiles between FAERS and JADER underscores how regional surveillance architectures shape risk identification. FAERS emphasized behavioral and iatrogenic risks—exemplified by the pronounced signals for drug abuse (ROR 31.3) and administration errors (ROR 71.08)—reflecting Western preoccupations with prescription integrity and non-medical use. Conversely, JADER prioritized acute physiological compromise, with respiratory depression (ROR 57.41) and neonatal respiratory failure (ROR 212.77) dominating its signal spectrum. This dichotomy likely arises from systemic reporting biases: FAERS captures consumer-generated concerns about medication safety and misuse, while JADER’s physician-driven reporting reflects clinical vigilance toward acute organ toxicities in hospitalized settings. Such divergence impedes global risk assessment but also offers complementary insights, illustrating how cultural, regulatory, and healthcare delivery factors sculpt pharmacovigilance narratives.

Second, our multi-algorithm consensus approach identified novel, clinically significant safety signals with plausible biological mechanisms. Device-dependent complications—transdermal adhesion failure (ROR 71.08), leakage, and application site reactions—dominated FAERS reports, implicating formulation-specific risks. These may result from a combination of factors: the local high concentration of fentanyl and its excipients causing direct tissue toxicity or irritation; occlusive effects of the patch leading to changes in skin microbiome and barrier function; and potential thermal amplification under the patch covering. These findings align with case studies describing fatal aspiration pneumonitis following accidental patch ingestion and chronic inflammation at application sites, suggesting excipient-mediated tissue toxicity (Bansari et al., 2024; Armenian et al., 2018; Ferdosnejad et al., 2025). Meanwhile, JADER revealed severe autonomic crises, including Kounis syndrome (allergic coronary vasospasm) and pulmonary hypertensive crisis, potentially mediated by mast cell degranulation and endothelial dysfunction triggered by fentanyl’s direct action on opioid receptors in vascular smooth muscle. Opioid receptors, particularly μ-receptors, are expressed on mast cells. Fentanyl binding may trigger mast cell degranulation, releasing histamine, leukotrienes, and other inflammatory mediators that induce coronary vasospasm and plaque destabilization. Similarly, signals for pulmonary hypertensive crisis could be related to opioid-induced histamine release causing pulmonary vasoconstriction, or a direct effect on pulmonary vascular tone (Yasunaga et al., 2015; Leo et al., 2021). The alarming disproportionality of neonatal morbidity signals (respiratory failure, withdrawal syndrome) corroborates clinical evidence of fentanyl’s placental transfer and immature hepatic metabolism, exacerbating respiratory depression in newborns. Among these, the strong signal for neonatal respiratory failure (ROR = 212.77) and other morbidities can be explained by the unique developmental pharmacology of neonates. The neonatal blood-brain barrier is not fully formed, and the hepatic cytochrome P450 system (particularly CYP3A4, crucial for fentanyl metabolism) exhibits markedly low activity. This leads to significantly prolonged fentanyl elimination half-life and increased systemic exposure, rendering neonates exquisitely sensitive to even therapeutic doses, culminating in respiratory depression (Liu et al., 2025; Temple et al., 2025). These signals collectively highlight underrecognized pathophysiological pathways extending beyond μ-opioid receptor activation, warranting mechanistic studies on mitochondrial dysfunction, neuroinflammation, and immunomodulation.

Third, demographic and temporal stratification exposed modifiable risk patterns critical for precision risk mitigation. Gender disparities revealed females consistently vulnerable to therapeutic failure (drug ineffective; ROR 1.22), application site reactions (pruritus; ROR 2.17), and gastrointestinal events. This profile aligns with known sex differences in opioid pharmacokinetics: reduced CYP3A4 activity and enhanced µ-opioid receptor density in females may prolong drug exposure and amplify somatic sensitivity (Oto et al., 2021; Zhu, 2025). Conversely, males exhibited heightened susceptibility to intentional misuse (abuse; ROR 0.26) and overdose, reflecting psychosocial and neurobiological factors, including greater risk-taking behavior and attenuated κ-opioid receptor-mediated dysphoria (Fitzgerald et al., 2025b). The bimodal temporal hazard—51% of ADEs within 30 days versus 23% > 1 year post-initiation—reveals distinct risk epochs: early events likely stem from titration errors and individual susceptibility (e.g., respiratory depression in opioid-naïve patients), while delayed complications reflect cumulative toxicity (Williamson and Kermanizadeh, 2024) (e.g., hypogonadism, neuroadaptation) or chronic device exposure. This decreasing hazard profile (Weibull β = 0.43) underscores the need for dynamic monitoring strategies, with intensive surveillance during initiation and long-term vigilance for endocrine and neurological sequelae.

The distinct temporal pattern of fentanyl-associated adverse events, characterized by a high initial hazard (Weibull β < 1) with 51% of events occurring within 1 month and a substantial proportion (23.1%) emerging after 1 year, carries direct implications for risk mitigation in clinical practice. This supports the implementation of a biphasic monitoring strategy. During the high-risk initiation phase (the first 30 days), vigilance should be greatest. For opioid-naïve patients, this warrants close observation for early signs of respiratory depression and sedation. Equally important is comprehensive patient education upon treatment initiation, focusing on proper administration techniques for transdermal patches to prevent errors and accidental exposure, and recognition of early adverse effects. Conversely, the persistence of risk beyond 1 year underscores the need for structured long-term vigilance during the maintenance phase. Clinical follow-up should include regular dermatological assessments for patients using transdermal systems, monitoring for potential endocrine sequelae such as hypogonadism, and repeated evaluation of behavioral risks including misuse or addiction. This temporal risk stratification enables a more efficient allocation of clinical resources, ensuring intensive support when risk is highest while maintaining necessary surveillance for delayed complications throughout therapy.

Consistent with previous systematic reviews and drug safety analyses, our data reaffirm well-established risks of respiratory depression and misuse associated with mu-opioid receptor activation (Volkow, 2021; Han et al., 2022; Li et al., 2024). However, our integrated multinational analysis provides critical advancements that extend the current evidence base. First, whereas prior pharmacovigilance studies were often limited to single center (Rahman et al., 2022), our parallel analysis of both systems not only confirms trans-regional concerns like withdrawal syndrome but, more importantly, reveals how geographic disparities and reporting practices shape distinct safety profiles—such as the heightened signal for respiratory depression in JADER, a pattern underrecognized in FAERS-centric literature. Second, moving beyond the general opioid toxicities commonly reported, our study detects novel and severe associations including Kounis syndrome and neonatal respiratory failure, which imply pathophysiological pathways beyond classical opioid receptor mediation. Third, by integrating a multi-method consensus approach (ROR, PRR, BCPNN, MGPS), we enhance the robustness of signal detection, particularly for rare, device-specific, and delayed-onset events—such as transdermal patch adhesion failure and application-site injuries—that are seldom captured in clinical trials or single-method disproportionality analyses. Thus, our work moves beyond confirming established risks to delineating a more complex, formulation-dependent, and demographic-aware safety profile, thereby addressing material gaps in the real-world pharmacovigilance literature.

### 4.1 Clinical and regulatory implications

The comprehensive safety profile of fentanyl elucidated in this study—characterized by novel signals related to transdermal delivery systems, neonatal morbidity, and pronounced geographic disparities—necessitates a concerted global response from regulatory bodies. Transdermal systems were implicated in over half of application site reactions in FAERS, indicating critical deficiencies in real-world product performance and patient guidance (Supplementary Table S3). Concurrently, strong signals of neonatal respiratory failure (ROR = 212.77) and withdrawal syndrome underscore vulnerabilities resulting from efficient placental transfer and immature neonatal metabolism (Wanar et al., 2023). Furthermore, divergent signal profiles between FAERS and JADER—such as the heightened reporting of respiratory depression in Japan—reveal systemic surveillance biases that may obscure globally relevant risks (Volkow et al., 2023).

Building on this evidence, we propose the following targeted recommendations for key regulatory agencies, while drawing insights from international practices such as China’s integrated approach to fentanyl control.

For the U.S. Food and Drug Administration (FDA), the prominent signals related to drug abuse, administration errors, and transdermal device failures call for enhanced risk evaluation and mitigation. We recommend: ①Updating the boxed warning to explicitly highlight risks of misuse, accidental exposure, and application site injuries. ②Mandating post-marketing studies of the FD&C Act to evaluate adhesion performance and skin tolerance under real-world conditions, with sex-stratified analysis. ③Strengthening the Risk Evaluation and Mitigation Strategy (REMS) to include mandatory prescriber education on abuse recognition and patient counseling on safe storage and disposal.

For the European Medicines Agency (EMA), the focus should be on quality oversight and proactive signal management: ①Enhancing quality assessment of transdermal systems by requiring rigorous human factors data on adhesion stability and drug release under variable environmental conditions as part of marketing authorization applications. ②Incorporating quantitative benefit-risk methods into Periodic Safety Update Reports (PSURs) to formally evaluate emerging signals such as Kounis syndrome. ③Adding severe autonomic reactions and neonatal respiratory depression to the list of “additional monitoring” features in product information.

For Japan’s Pharmaceuticals and Medical Devices Agency (PMDA), strong signals for acute respiratory depression, delirium, and neonatal morbidity warrant clinical guidance and vigilant surveillance: ①Revising package inserts to emphasize risks of respiratory and neurological events, particularly in elderly and comorbid patients. ②Implementing a standardized MedDRA query for device-related adverse events to improve signal detection within JADER. ③Requesting focused post-marketing surveillance on neonatal outcomes and cardiovascular events from marketing authorization holders.

Globally, we advocate for harmonization initiatives to strengthen pharmacovigilance infrastructure: ①Developing standardized MedDRA terminology for device- and formulation-specific adverse events to enable cross-database comparison. ②Promoting linked mother-infant dyad reporting to improve capture of long-term developmental outcomes.

These collective findings also support tailored clinical monitoring strategies, including enhanced respiratory observation in neonates, dermatological evaluation in females using transdermal systems, and standardized substance use screening in male patients (Jeffery et al., 2023; Hendrickson et al., 2025). The pronounced hazard peak during the initial 30 days of therapy further underscores the need for structured education and early clinical surveillance during treatment initiation (Reyes et al., 2025). Future pharmacovigilance efforts should integrate complementary real-world data sources, such as electronic health records and specialized registries, to corroborate signals and enhance cross-population generalizability.

### 4.2 Limitations and future directions

While our integrated pharmacovigilance study provides novel insights into fentanyl safety, several limitations must be acknowledged.

First, as with all analyses of spontaneous reporting systems, our study is susceptible to inherent biases, including under-reporting, over-reporting of severe events, and the absence of a definitive denominator, which precludes the calculation of incidence rates. Second, the data granularity is limited; reports often lack detailed clinical information (e.g., precise dosing, concomitant medications, and underlying patient comorbidities), which restricts the ability to control for potential confounding factors. Third, despite integrating two major databases, geographic coverage remains skewed toward the United States and Japan, potentially limiting the generalizability of findings to other regions with different medical practices and opioid prescribing patterns. Finally, and most importantly, disproportionality analyses can detect statistical associations but cannot establish causality; the signals identified herein require validation through well-designed analytical epidemiological studies.

Looking forward, these limitations outline a clear agenda for future research. Prospective studies are needed to corroborate the novel signals we detected, such as Kounis syndrome and application site injuries. Future frameworks should strive to integrate SRS data with complementary real-world data sources, such as electronic health records and claims databases, to obtain denominator data and better control for confounding. Furthermore, incorporating multi-omics biomarkers (e.g., pharmacogenomic predictors of respiratory depression) and utilizing AI-enhanced pharmacovigilance platforms could enable real-time signal detection and a more personalized assessment of fentanyl-related risks, ultimately bridging the translational gap between signal generation and clinical practice.

## 5 Conclusion

Fentanyl’s safety profile extends far beyond classical opioid toxicology, encompassing device-dependent failures, demographic-specific vulnerabilities, and delayed systemic complications. Our analysis provides a roadmap for targeted risk minimization: prioritizing transdermal system redesign, sex-specific dosing guidance, and neonatal exposure protocols. Crucially, it advocates for pharmacovigilance systems that reconcile regional reporting disparities through shared data standards and proactive surveillance of novel toxicological mechanisms. Only such integrated approaches can balance fentanyl’s indispensable analgesic benefits against its multifaceted harms in an evolving opioid landscape.

## Data Availability

The original contributions presented in the study are included in the article/Supplementary Material, further inquiries can be directed to the corresponding author.
